# Non-pharmacological Treatment of Refractory Angina and Microvascular Angina

**DOI:** 10.3390/biomedicines8080285

**Published:** 2020-08-13

**Authors:** Kudrat Rakhimov, Tommaso Gori

**Affiliations:** 1 Department of Cardiology, University Medical Center Mainz Langenbeckstr 1, 55131 Mainz, Germany; 2 Department of Cardiology, University Medical Center Mainz and Deutsches Zentrum für Herz und Kreislauf Forschung, Standort Rhein-Main, Langenbeckstr 1, 55131 Mainz, Germany

**Keywords:** refractory angina, microvascular angina, microvascular dysfunction

## Abstract

Refractory angina (RA) is defined as debilitating anginal symptoms despite the optimal guideline-directed combination of medical, percutaneous, and surgical therapies. Often referred to as “no option”, these patients represent a significant unmet clinical need for healthcare institutions. Due to the ageing of the population, and increased survival from coronary artery disease, the number of patients with RA is expected to rise exponentially. Despite the developments of novel technologies for the treatment of RA, none of them found wide clinical application (to date). Microvascular dysfunction, alone or in combination with epicardial coronary disease, is thought to contribute significantly to refractory angina. However, most of the techniques developed to improve RA symptoms have not been tested specifically on patients with microvascular dysfunction. This review discusses the recent developments in the treatment of RA, and gives some perspectives on the future of these techniques.

## 1. Introduction

The expression refractory angina (RA) refers to the presence of angina symptoms, usually for ≥3 months which cannot be controlled by means of established pharmacological therapy, coronary bypass grafting or percutaneous interventions, including chronic total occlusions [1,2] Although the presence coronary artery disease (CAD), intended as obstructions of epicardial coronaries, is usually considered a prerequisite, RA may follow a wide range of clinical entities, including obstructive CAD, a microvascular disease with patent epicardial coronary arteries, hypertrophic cardiomyopathy, and left ventricular diastolic dysfunction [3]. Patients with RA tend to suffer from poor health status, psychological distress, and a reduction in quality of life, while their frequent symptoms place a considerable burden on healthcare resources.

Data from several epidemiological studies report that 5% to 10% (7.7% women, 7.3% men) of the patients with stable CAD undergoing cardiac catheterization have refractory angina, and the annual incidence of RA in Europe estimated to be 30,000–50,000 and 75,000 in the United States [4]. However, this data might be unreliable and outdated for several reasons: Firstly, many studies did not include patients with no epicardial obstructive CAD and microvascular angina. These clinical entities may account for a significant proportion of RA patients [5,6,7]; second, due to the developments in technical aspects of revascularization techniques and aging population, the real numbers may be rapidly changing. Relatively high survival rate at nine years (77.8%) suggests that clinicians will deal with an increased number of patients with long life expectancy and poor quality of life [8].

Despite the growing numbers of CAD patients with limited revascularization options or “no option”, only two new therapies have been approved for the treatment of RA in the last 40 years. Ranolazine was approved based on studies in stable CAD patients on single or no medical therapy and enhanced external counterpulsation demonstrated improvement in time-to-ST depression, but not overall exercise capacity in a RA population. However, more recently, the effectiveness of ranolazine were controversial in both large randomized trial [9] and small-scaled registry [10]. Other techniques, such as coronary sinus reducer [11] and extracorporeal shock wave therapy [12], have shown therapeutic potential, although none of them has so far gained enough acceptance to enter the routine therapeutic armamentarium. 

In this review, we set out to describe the current state of non-pharmacological techniques for patients with refractory angina and a subset of patients with microvascular dysfunction, for which treatment options are particularly scarce (Table 1).

## 2. Coronary Sinus Reducer

**Figure 1:**
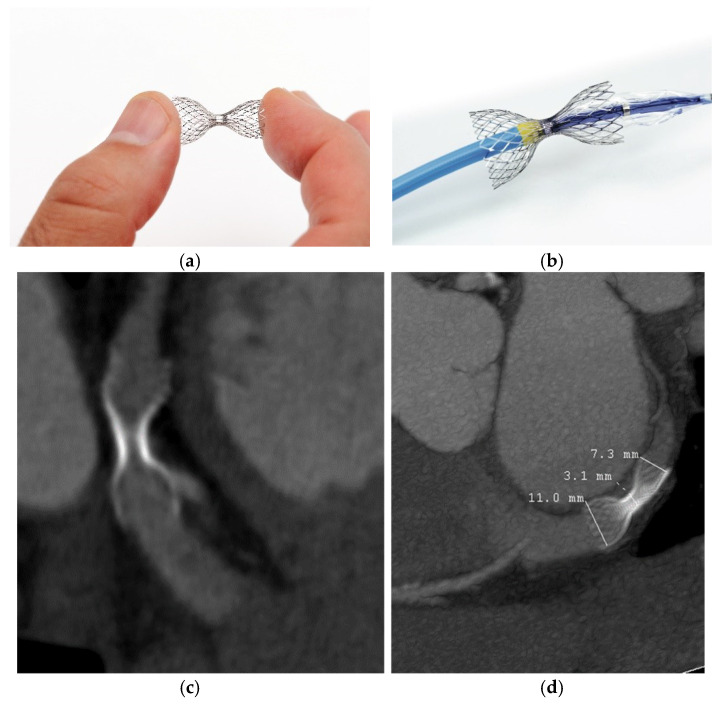
The Sinus Reducer Device (Neovasc Inc. Richmond, Canada) is a stainless steel, hourglass-shaped, balloon-expandable mesh designed to create focal luminal stenosis in the coronary sinus (top left and right). Computer tomography image of the coronary sinus after 1-year follow-up (bottom left and right). (**a**) The sinus reducer; (**b**) the sinus reducer as mounted on its delivery balloon. (**c**,**d**) computer tomography after implantation.

The concept of coronary sinus pressure augmentation was pioneered and first performed surgically by Beck et al. in the mid-1950 s [19]. Experimental narrowing of the coronary sinus through partial ligation led to infarct size reduction and symptom relief in patients with severe CAD. This work paved the way for the future development of percutaneous techniques—the coronary sinus reducer (CSR) devices. The CSR is a stainless steel, hourglass-shaped, balloon-expandable mesh designed to create focal luminal stenosis in the coronary sinus (Figure 1). The device is implanted percutaneously via a jugular vein under the local anesthesia. The narrowing can take up to six weeks before the device is completely endothelialized [20,21].

In a healthy heart, exercise or increased demand is compensated by sympathetically mediated vasoconstriction of subepicardial arteries, favoring blood flow to subendocardial vessels in order to contrast the increase in left ventricular end-diastolic pressure, maintain perfusion and preserve myocardial contractility. In patients with advanced CAD, however, this compensatory mechanism is compromised and during exercise, blood flow is redistributed towards the subepicardial vessels, causing impaired contractility and ischemia. These mechanisms explain the typical ischemic susceptibility of the subendocardial territories to increases in oxygen consumption. As a further complication, in cases of increased afterload, systolic or diastolic left ventricular dysfunction, increased left ventricular end-diastolic pressure (LVEDP) will compress subendocardial vessels, further contributing to ischemia [22,23]. Bearing in mind the above, the physiological rationale for CSR use is the hypothesis that the increased backpressure in coronary sinus through partial occlusion would redistribute collateral flow from the less ischemic epicardium to the ischemic endocardium as demonstrated by Stoller et al. [24] in a study in 35 patients with CAD. In this mechanistic study, the authors report that the coronary collateral flow index (calculated as the ratio between coronary pressure distal to a balloon and aortic pressure) improved along with less ECG evidence of ischemia during experimental simultaneous coronary sinus and coronary artery occlusion as compared to coronary artery occlusion alone. The hypothesis that coronary sinus occlusion might also improve coronary perfusion in the microcirculation is supported by animal studies and preliminary human evidence. Ido et al. demonstrated that the increase in venous pressure leads to dilatation of the subendocardial arterioles, resulting in a significant reduction of vascular resistance in this area and a redistribution of blood flow to these ischemic subendocardial layers [11]. In a small case series by Giannini et al., patients with microvascular angina showed clinical improvement after CSR [25]. Finally, preliminary data from our laboratory appear to support the concept that coronary sinus occlusion might improve microvascular resistances: In a recently published case report [26] we observed a ~50% decrease in resting and hyperemic resistances in a patient tested before and after coronary sinus occlusion. Although these are just initial data, they are compatible with the concept that the redistribution of blood from the subepicardial to the subendocardial space might be associated with a drop in total microvascular resistances (and therefore, relief of symptoms).

The safety of the CSR device was demonstrated in the first-in-man study of CSR implantation, with no periprocedural and 12 months major adverse cardiac events [20]. Although underpowered for efficacy, sustained improvement in the CCS angina class was documented at six months and three years [27]. Expanding on this evidence, the single double-blind, sham-controlled trial to date, COSIRA (Coronary Sinus Reducer for Treatment of Refractory Angina) trial [13], randomly assigned 104 RA patients in a 1:1 fashion to CSR implantation or sham procedure. The primary end-point (a reduction of ≥2 CCS classes at six months) occurred ≈2.5 times as frequently in the treatment group as in the control group, 35% vs. 15% (*p* = 0.024). Assessed as secondary efficacy end-points (however, not powered), symptom relief at least I CCS class was reported in a greater proportion of patients (71% vs. 42%, *p* = 0.003); improvement in the quality of life assessed by the Seattle Angina Questionnaire was also significant. 

Real-world data from several centers have been collected in recent years, further supporting the safety and efficacy of the procedure, with a high success rate (≥98%), no device-related adverse events, and high rate of responders (≥70%) at one and two years [28,29,30,31,32,33]. Along with its symptom-relieving effect CS narrowing has also been shown to improve objective indices of ischemia (dobutamine echocardiography) and physical function (6-min walk test, treadmill ergometry, cardiopulmonary exercise test) [30,32]. Moreover, several observational studies [34,35] and case-reports [36,37] documented left ventricular function improvement using stress magnetic resonance imaging after CSR implantation, especially in patients with reduced LVEF. These findings led to the speculation about the physiological rationale to test CS narrowing in ischemic cardiomyopathy. There were concerns regarding this technology that the elevated venous pressure and consequent myocardial interstitial stasis would cause diastolic dysfunction. However, recently single-center observational study, including 24 RA patients undergoing CSR implantation reported improved echocardiographic diastolic parameters at six months [38]. A phase III multicenter, randomized, double-blind, sham-controlled trial (COSIRA-II), which is planned to enroll 380 RA patients and in 35 centers in the USA and Canada, will hopefully shed light on the efficacy and safety of the technique [39].

## 3. Revascularization of Chronic Total Occlusions 

Revascularization by the intervention of chronic total occlusions (CTO) is another option in RA patients with documented occluded coronary arteries [40]. Despite increased success rate in recent years [41], this method remains lengthy, technically challenging, contrast-consuming, not devoid of complications and requires skilled operators. As for the other options discussed in the present review, it remains unclear whether CTO interventions offer prognostic improvement or rather only improve angina symptoms. Data from observational studies show clinical benefit and quality of life improvement following successful treatment of CTOs [42,43]. In contrast, the evidence on the prognostic benefit of these complex procedures is less clear: While a recent meta-analysis showed improved survival in patient following a successful treatment as compared to patients with a failed attempt [44], data from randomized trials confirmed an improvement in angina frequency and quality of life, but showed no effect in overall mortality [45,46]. Given these limitations, the selection of patients and indication for such procedures have a major role in the decision to perform a CTO recanalization procedure. It would go beyond the scope of the present review to discuss the indications, methods and technologies used in CTO interventions. The latest European Guidelines on myocardial revascularization, however, recommend CTO procedures with a class Iia (Level of evidence B) as long as evidence of vitality and ischemia, as well as a reasonable expectation that the procedure will lead to ischemia reduction, are present [47]. 

With these limitations in mind, CSR might remain a valid alternative in patients with CTO lesions. A recent retrospective study showed greater symptom improvement (CTO 80.6% vs. no CTO 66.3%, *p* = 0.03) in RA patients with evident CTO lesions as compared to patients without CTO lesions [48]. Interestingly, symptoms improvement was also evident in patients with isolated right coronary artery (RCA) CTOs. Venous drainage of this artery is independent of the coronary sinus, which could explain the lack of CSR efficacy in RCA-related areas. However, this hypothesis should be tested in large randomized trials.

## 4. Enhanced External Counterpulsation

**Figure 2:**
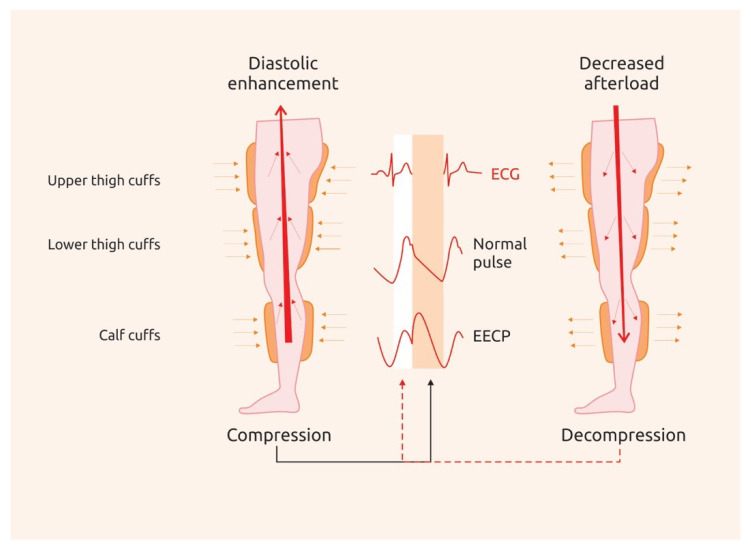
Principles of enhanced external counterpulsation (EECP). EECP produces a diastolic retrograde aortic flow that enhances coronary artery mean and peak diastolic pressure by sequential compressions and decompressions of the three pairs of cuffs (upper thigh, lower thigh and calf).

Enhanced external counterpulsation (EECP) is a non-invasive technique which has been shown to improve coronary perfusion by inducing arterial retrograde flow during diastole. Beyond its afterload-reducing effects, which would be expected to be analogous to those of intra-aortic balloon pump, EECP also increases venous return to the heart. EECP consists of sequential compressions at 300 mm Hg and decompressions of three pairs of cuffs placed around calves, lower and upper thighs. ECG-gated compressions take place in early diastole in a distal-to-proximal sequence and decompressions occur just before systole (Figure 2). Thirty-five sessions administered as 1 h per day over seven weeks are needed in order to achieve the desired effect.

Although the mechanisms by which EECP exerts its antianginal effect are not completely understood, several studies gave insights into possible mechanisms. Increased collateral arteriogenesis and improved coronary flow reserve by enhanced nitric oxide production and decreased endothelin-1 have been postulated as the main mechanism of EECP [49,50,51]. EECP has also been shown to improve endothelial function [52], to reduce arterial stiffness [53] and inflammatory cytokine levels [54], to induce peripheral flow-mediated dilatation [55] and to increase circulating progenitor (CD34+) cells [56].

Due to the nature of the procedure, double-blind controlled trials are impossible to carry out, which raises questions regarding an operator bias and/or a placebo effect. To date, the only double-blind sham-controlled trial to date, the multicenter study of enhanced external counterpulsation (MUST-EECP) trial, which included 139 patients angina and documented ischemia, randomized patients to active EECP and hemodynamically inactive counterpulsation [14]. Patients in EECP group experienced fewer angina symptoms (*p* < 0.09) and showed the improved time to ≥1-mm ST-segment depression on the treadmill (*p* = 0.01). It is worth mentioning that the MUST-EECP trial, like many other trials, did not include RAP patients specifically. Two meta-analyses reported improvement in at least one CCS angina class in 85% [57] and 86% [58] of RA patients. Another meta-analysis, including six prospective studies, showed increased myocardial perfusion in patients with CAD [59].

Despite its high safety profile, EECP has several contraindications, such as severe valvular heart disease (especially aortic insufficiency), arrhythmias, coagulopathy with INR > 2.5, severe peripheral artery and venous disease, decompensated heart failure and severe hypertension (>180/110 mm Hg). Finally, despite accumulated evidence on efficacy, safety and cost-effectiveness [60], EECP has not yet been fully translated into clinical use owing to its time-consuming regimen and to the lack of specialized centers.

## 5. Extracorporeal Shockwave Myocardial Revascularization

Extracorporeal shockwave myocardial revascularization (ESMR) is another promising non-invasive technique aimed at improving myocardial perfusion in ischemic areas through the application of acoustic waves [12]. ESMR applies low-intensity shockwaves (0.09 mJ/mm^2^, one tenth of all energy delivered in lithotripsy) with a resolution of millimeters to any intended treatment area under echocardiographic guidance. Nine 20-min treatment sessions consisting of 100 shocks per treatment zone over nine weeks are recommended according to the treatment protocol [61]. ESMR is a safe and well-tolerated procedure, with bad acoustic window and left ventricular thrombus being the contraindications. 

**Figure 3:**
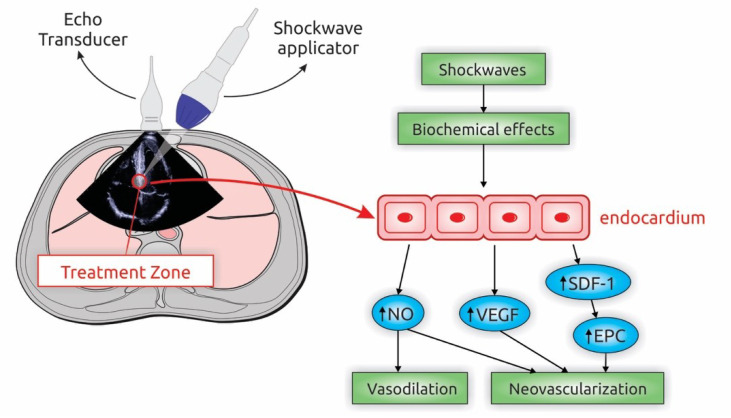
Schematic Presentation of the Extracorporeal Shockwave Myocardial Revascularization (ESMR) and putative mechanism of action. ESMR improves myocardial perfusion through the application of low-intensity shockwaves to ischemic areas. Shear stress on endocardial cell membranes, induced by shockwaves, is thought to lead to hyperpolarization, activation of Ras and formation of free radicals, with consequent enhancement in nitric oxide production (NO), up-regulation of chemoattractants, such as vascular endothelial growth factor (VEGF), stromal-derived factor-1 (SDF-1). These chemoattractants, in turn, can lead to progenitor cells recruitment to the ischemic tissue areas. EPC, endothelial progenitor cells.

Several angiogenic pathways have been suggested to be involved in the beneficial effects of ESMR [62,63]. Shear stress on endocardial cell membranes, induced by shockwaves, is thought to lead to hyperpolarization, activation of Ras and formation of free radicals, with consequent enhancement in nitric oxide production [64], up-regulation of chemoattractants, such as vascular endothelial growth factor [65,66,67], stromal-derived factor-1. These chemoattractants, in turn, can lead to progenitor cells recruitment to the ischemic tissue areas [67]. Thus, ESMR may lead to vasodilation, reduced inflammatory response and neovascularization (Figure 3). 

A data from both small randomized-controlled trials and real-world experiences confirmed the efficacy of ESMR in terms of symptom improvement, reduced use of short-acting nitrates and hospitalizations, increased myocardial perfusion at different follow-up periods up to 72 months in patients with stable CAD and specifically RA patients [66,67,68,69,70,71]. A prospective controlled study of 72 RA patients showed improved symptoms and quality of life parameters, increased myocardial perfusion at six months [67] with sustained effect at a longer time period (2.88 ± 1.65 years) [68]. Results of a meta-analysis, including 39 studies, 1006 patients were in line with the previous studies of ESMR, confirming the efficacy and safety of the strategy [72]. It is noteworthy that this meta-analysis included both randomized-controlled and prospective single-arm studies, with the largest randomized trial, including 45 patients. However, despite recent developments, ESMR remains an experimental tool and larger multi-center, adequately powered randomized double-blind trials are warranted before its wide clinical application.

## 6. Stem Cell Therapy

Cell therapy with bone marrow (BM)-derived progenitors has emerged as a promising therapeutic option for RA patients. Different BM autologous vasculogenic cell populations, including unfractioned mononuclear cells, CD34+ or CD133+ cells, have been injected into ischemic areas to ameliorate perfusion of LV territories not otherwise amenable to revascularization [73]. There are two most common delivery routes of these cells: Intra-myocardial and intra-coronary. Circulating levels of CD34+ cells predict advanced CAD, physical function, adverse clinical outcomes after myocardial infarction, and overall survival [74,75]. In preclinical myocardial infarction models, isolated CD34+ cells were associated with improvements in overall myocardial performance and regional wall motion, and they showed potential in reducing fibrosis and increasing angiogenesis [76]. 

Safety and feasibility of intramyocardial injection of autologous CD34+ stem cells were demonstrated in a Phase I/IIa double-blind, randomized controlled trial on 24 RA patients, showing potential bioactivity with improvement in CCS angina class [15]. A phase II randomized trial, including 167 RA patients, showed a significant improvement in both angina symptoms and exercised tolerance with intramyocardial injection of autologous CD34+ stem cells over placebo at 6, 12 months [77] with persistent results at 24 months follow-up [78]. In addition, there was a trend towards a reduction in the rates of major adverse cardiac events (*p* = 0.08) [78]. Based on these promising results, a phase III randomized (RENEW, efficacy and safety of targeted intramyocardial delivery of auto CD34+ stem cells for improving exercise capacity in subjects with refractory angina) trial was launched. However, the trial was terminated prematurely by the sponsor, due to financial reasons, recruiting only 112 patients of planned 444, which was not sufficient to test its efficacy end-point [16]. Pooled analysis of the three previous trials, including a total of 276 patients demonstrated sustainable improvements in exercise capacity, angina frequency and mortality at 3, 6, and 12 month-time points [16]. The safety and efficacy of intracoronary injection of autologous CD34+ stem cells in two concentrations (low-dose and high-dose) were evaluated in 38 patients with no option CAD and left ventricular dysfunction in a phase I randomized trial [79]. The main findings of the trial were improved angina, left ventricular ejection fraction, reduced remodeling at one and five years follow-up [80,81]. Intriguingly, improved clinical outcomes and heart function were correlated with angiogenesis assessed by coronary angiography, rather than CD34+ dose. 

Trans-endocardial delivery of another autologous bone marrow-derived cells, CD133+ cells, were proved to be safe and feasible in two small phase I randomized trials not powered for efficacy end-points [82,83]. Another recent phase I randomized trial of intramyocardial injection of CD133+ cells in RA patient subset with LV dysfunction (LV < 45%) confirmed the safety profile of the previous two trials and showed significant improvements in CCS angina class, myocardial perfusion and function assessed by single-photon emission computer tomography at 12 months [84]. Moreover, improvements in myocardial perfusion were positively correlated with the proangiogenic growth factors, hepatocyte growth factor and platelet-derived growth factor type bb, substances involved in neovascularization, endothelial and muscle cell growth [85,86]. The most recent and the largest (8 RCTs, 526 patients) meta-analysis to date concluded that cell-based therapies improve not only indices of angina (angina episodes, Canadian Cardiovascular Society angina class, exercise tolerance, and antianginal medications), but also mortality and major adverse cardiovascular events [87,88].

Despite recent advancements in this field, questions regarding optimal cell type, dosage, delivery method duration of efficacy remain open. Thus, larger Phase III studies are needed to address these issues, assessing both clinically relevant outcomes (quality of life, cost-effectiveness and MACE) and quantitative assessment of myocardial perfusion (PET and cardiovascular magnetic imaging).

## 7. Spinal Cord Stimulation and Transcutaneous Electrical Nerve Stimulation (TENS)

The use of spinal cord/nerve stimulation for the treatment of RA bases on an alternative explanation for the source of the chest pain, i.e., that its cause might not reside within the myocardium, but rather originate from its elaboration within the somatosensory nervous system [89]. The central nervous system (CNS) is responsible for the perception of visceral chest pain, and it has been shown that patients who have been diagnosed with angina in the absence of coronary artery obstructions present characteristic regional differences in brain activation as compared to patients with ”traditional” CAD [89,90]. This appears to suggest that while angina in patients with CAD group is due to a supply–demand mismatch, the chest pain of patients with no CAD may be due to abnormal CNS processing of afferent signals from the myocardium, leading to inappropriate and increased cerebral cortex activation and the subjective feeling of pain. SCS has been designed to correct this maladaptive neuropathic process, even though additional myocardial effects have also been demonstrated: In a study of 60 patients undergoing coronary angioplasty, de Vries et al. investigated the effect of transcutaneous electrical neurostimulation on functional collateral perfusion, assessed as the ratio of coronary wedge pressure to aortic pressure [91]. Using a cross-over design in two parallel groups, the authors show that the Pw/Pa ratio increases during ischemia when electrical neurostimulation is active, while it decreases when it is inactive, an effect that might be due to collateral vessel recruitment via b-adrenergic receptors. Finally, some studies showed a reduction in afterload and systemic vasodilatation following SCS, possibly due to reduced sympathetic activity. 

SCS is a surgical procedure that consists of implantation of one or more leads in the epidural space of the spinal canal. The lead(s) are connected to an implantable pulse generator and delivers on-demand a weak electrical current to the spinal cord, resulting in peripheral paresthesia and withdrawal of pain. Beyond RA, SCS is used for complex regional pain syndrome and radicular pain after failed back surgery syndrome, stump pain after amputation, and pain due to peripheral nerve injury, peripheral vascular disease, and diabetic neuropathy. Symptomatic success rates for these indications have been reported to be in the range of 50–75%. 

Studies on SCS in RA are mainly limited to small, open-label studies [92,93,94,95]. The largest blinded study to date, STARTSTIM [17], was underpowered, due to slow patient enrollment and subsequent early termination. The analysis of 68 patients failed to show the efficacy of SCS against control-group, showing significant improvement in both groups consistent with the placebo effect. Two recent meta-analyses of 14 [96] and 12 [97] studies in RA patients showed longer exercise duration, lower angina frequency and nitrate consumption associated with SCS. Future studies will have to test whether these effects are confirmed in larger cohorts. 

## 8. Transmyocardial Laser Revascularization

Transmyocardial laser revascularization (TMLR) is a laser technique intended to create 1 mm channels within the wall of the left ventricle, a procedure which can be performed either percutaneously or surgically through lateral thoracotomy under general anesthesia. The rationale behind this procedure and the mechanisms of its putative effectiveness remain unclear, and the hypothesis that the channels provide direct myocardial perfusion proved unreasonable, as the channels were shown to close after few days of the procedure. Two other mechanisms, including denervation and angiogenesis, have been proposed, but no clear evidence persists [98].

Despite the encouraging results of efficacy and safety reported in initial studies, TMLR has been subject to substantial controversy, due to the lack of consistency in the following studies and the lack of explanation about the possible mechanisms of action. A meta-analysis of 10 trials and 1359 patients comparing TMLR against medical therapy (7 trials), CABG (2 trials) or sympathectomy (1 trial) reported unfavorable results in terms of risk/benefit ratio, despite improvements in subjective outcome measures, such as angina score, exercise tolerance and quality of life questionnaire [98]. Another meta-analysis of seven trials comparing TMLR with medical therapy further confirmed the findings of the previous analysis [99].

Several laser types and techniques have been developed using a percutaneous approach. The only double-blinded randomized trial in 298 using a Holmium: YAG laser showed no benefits of both doses (high-dose, low-dose) of percutaneous TMLR compared to a sham procedure at six months, rather reporting more acute MI cases with this technique [18]. A meta-analysis by the National Institute for Health and Care Excellence reported no benefits of percutaneous TMLR over a control group in terms of CCS angina class, exercise tolerance and myocardial perfusion at 12 months [99]. Taking into consideration the fact that no new trials on TMLR, both surgical and percutaneous, have been published in the last fifteen years, the procedure seems to become outdated.

## 9. Refractory Microvascular Angina

It has been suggested that microvascular dysfunction (MVD) is a significant component contributing to RA [5,100,101,102]. Despite the optimal guideline-directed pharmacological and life-style interventions, more than half of the patients (55%) with microvascular angina (RMVA) have refractory symptoms that are not alleviated by medical therapy [103]. However, treatments options in this cohort of patients remain particularly limited. Moreover, as MVD is not tested routinely during invasive coronary angiography, it remains largely underdiagnosed [104]. Therefore, treatment of RA should not focus only on the macro-, but also the microvascular dysfunction.

The utility of several new therapeutic tools have been tested in the management of RA in the last three decades, however, only a small portion of these were validated specifically on the population of patients with RMVA. Namely, EECP has been shown to improve coronary flow reserve (a marker of microvascular function) [105], index of microvascular resistance [106] along with improvements in CCS angina class and exercise tolerance [106,107,108]. These effects were thought to be mediated by stimulating new collateral artery formation and improved endothelial function. CSR implantation in eight patients with MVD in RA patients were associated with symptom improvement, increased exercise tolerance and quality of life, and myocardial perfusion reserve index on dipyridamole stress cardiac magnetic resonance [33], showing therapeutic potential in this challenging subgroup of patients.

Cell therapy is another promising option to address refractory microvascular angina. Bone marrow-derived progenitor cells have been demonstrated to promote neovascularization and consequently myocardial perfusion leading to increased contractility in in vivo models [109]. Although underpowered to address MVD, substudies of two randomized trials, REPAIR-AMI [110] and TOPCARE-AMI [111] has documented improved coronary flow reserve of the infarct-related artery with intra-coronary injection of bone marrow-derived progenitor cells in patients with acute myocardial infarction. Two ongoing studies, CLBS14 trial (NCT03508609) and CD34 trial (NCT03471611), designed to assess the efficacy of CD34+ cells specifically on MVD will hopefully give more plausible data on this disease entity with limited treatment options.

## 10. What Do Guidelines Say?

Recent European guidelines [2] have given CSR, EECP, and spinal cord stimulation IIb class of recommendation and level B evidence, while the TMLR is classified as a harmful procedure (recommendation class III). American guidelines [112], which were published in 2012 and updated in 2014, EECP, SCS and TLMR fall under IIb class recommendations. The absence of CSR in American Guidelines is explained by the fact that the results of a major CSR trial, COSIRA, were published later.

## 11. Conclusions

Considerable research has been done to develop novel treatments for patients with RA in the last three decades. However, most of these therapies have not been translated into routine clinical practice and remain experimental. The barrier in most cases is the lack of convincing evidence from randomized trials (CSR, ESMR, Cell therapy, SCS), and in other cases are the logistic issues (EECP) and safety concerns (TMLR). Furthermore, the data on the specific subgroup of patients with RMVA remains even more scarce. In order to overcome these obstacles and to gain widespread clinical acceptance, large randomized trials addressing the most important issues, including mechanism of action, efficacy, logistics and cost-effectiveness, must be sought.

## Figures and Tables

**Table 1:** Main characteristics of novel techniques in Refractory Angina.

	Mechanism of Action	Invasive-ness	Complications	Cost	RCTs	Guideline Recommendations
CSR	- Blood flow redistribution towards ischemic myocardium - Neovascularization - Improvement of microvascular function	+	Device migration (rare) Coronary sinus perforation, dissection, thrombotic occlusion (rare) Access site bleeding	Similar to CABG	COSIRA [13]	ESC (IIb/B), ACC/AHA (NA)
EECP	- Improved coronary diastolic perfusion - Neovascularization - Improvement of endothelial function - Reduced afterload	-	Paresthesia Leg pain Skin abrasion	Low	MUST-EECP [14]	ESC (IIb/B), ACC/AHA (IIb/B)
ESMR	- Improved coronary perfusion - Neovascularization	-	None	Low	NA	NA
Cell Therapy	- Neovascularization - Improvement endothelial function	+	Ventricular Tachycardia Access site bleeding Stroke (rare)	NA	ACT-34 [15], RENEW [16]	NA
SCS	- Reduced Heart Rate, Blood Pressure through sympathetic activity reduction - Improved myocardial perfusion	+	Lead migration Lead fracture Infection Headache Neurological damage	Similar to CABG	STARTSTIM [17]	ESC (IIa/B), ACC/AHA (IIb/B)
TMLR	- Angiogenesis - Direct myocardial perfusion	++	Myocardial infarction Cardiac tamponade Heart failure Death	High	[18]	ESC (III/A), ACC/AHA (IIb/B)

CSR, coronary sinus reducer; EECP, enhanced external counterpulsation; ESMR-, extracorporeal shockwave myocardial revascularization; SCS, spinal cord stimulation. TMLR, transmyocardial laser revascularization; RCT, randomized-controlled trial; ESC, European Society of Cardiology; ACC/AHA, American Society of Cardiology/American Heart Foundation; CABG, coronary artery bypass graft.
